# Views about Euthanasia and Dementia: Exploring Perceptions Utilising Evidence from the Mass Observation Archive

**DOI:** 10.3390/healthcare11182552

**Published:** 2023-09-15

**Authors:** Janet Blain, Dean Stevens, Louise Taylor, Paul Kingston, Geoffrey Watts

**Affiliations:** The Faculty of Health, Medicine and Society, University of Chester, Chester CH1 4BJ, UK

**Keywords:** dementia, euthanasia, assisted dying, voluntary assisted dying, medical assistance in dying, assisted suicide, capacity, legislation, Mass Observation Project

## Abstract

This paper contributes to the ongoing discussion in the United Kingdom regarding euthanasia and assisted dying, using data specifically related to individuals with dementia. A qualitative approach was taken with data captured via a set of written questions in the form of a Mass Observation Archive Directive. The respondents, known as Observers, provided written responses; there was no limit to the length of the responses and the Observers were able to provide as much or as little detail as they chose. The data were analysed thematically utilising NVivo software. One-hundred and seven responses were received, representing a range of beliefs, and with opinions regarding euthanasia and dementia with pro, anti, and uncertain views being expressed. Five main themes emerged during our data analysis: capacity, legislation, agency and personal philosophies, disquietude, and incumbrance. Consistent with previous research capturing public views regarding euthanasia and assisted dying for people with dementia, the findings suggest policy makers may wish to consult the British public regarding legislation regarding euthanasia and dementia.

## 1. Introduction

This paper contributes to the ongoing debate in the United Kingdom (UK) regarding euthanasia. “Euthanasia” originates from the Greek “eu” denoting “well” and “thanatos” meaning “death”, i.e., a good death. This paper utilises euthanasia as a generic term for “assisted dying”, “physician-assisted dying”, and other related expressions. The paper takes an UK-centric approach, utilising data from members of the British public which focuses on euthanasia for people with dementia (PWD), thus providing a lay perspective. The Campaign for Dignity in Dying [1] highlights “200 million people around the world have legal access to some form of assisted dying”. Switzerland has allowed assisted suicide since 1942 with the caveat the individual must self-administer the life-ending drugs. The Netherlands was the first country to decriminalise euthanasia [2], and other European countries, including Belgium, Luxemburg, Spain, and Portugal followed. The position in North America is less clear cut; in the United States of America, legislation varies by state, whilst, in Canada, the Medical Assistance in Dying [3] act encompasses the whole country [4]. The debate continues in other jurisdictions [4].

While there are different types of dementia, the disease is incurable and life limiting, impacting cognition and language skills and with associated behavioural changes as the disease progresses [5]. In January 2022, it was estimated 944,000 PWD lived in the UK [6], with a 2019 report for the Alzheimer’s Society projecting an increase to 1,590,100 individuals by 2040 [7].

Guidelines from the National Institute for Health and Care Excellence [8] indicate care for PWD should be person-centred, with PWD being involved in decisions about their care, and with any decisions made on their behalf adhering to best interest principles. Given the progressive nature of dementia, individuals may decide to be proactive and plan for their future as the disease progresses. One option individuals may consider is making an advanced care plan (ACP), which is defined as:

*“a process of formal decision making to help patients establish decisions about their future care which takes effect when they lose capacity to make decisions for themselves.”*

[9]

A systematic review of terminally ill individuals’ experiences of ACPs [10] proposes making an ACP promotes personal agency because patients are asserting control over their treatment and death. Although the timing of the decision to make an ACP was key, confronting and accepting the end of life was upsetting for some individuals. Consequently, the timing of ACPs is particularly relevant in the case of dementia, as there is a need to balance concerns about prematurely making an ACP versus leaving it too late in the disease trajectory when the PWD may have already lost the capacity to effectively participate in the process [11]. The Mental Capacity Act [12] defines people who lack capacity as:

*“a person lacks capacity in relation to a matter if at the material time he is unable to make a decision for himself in relation to the matter because of an impairment of, or a disturbance in the functioning of, the mind or brain.”*

ACPs appear to offer individuals control over their future care and medical treatment; however, these are not legally binding [13]. Nonetheless, NICE guidelines (2019) [14] advise *“People with dementia are given the opportunity to discuss advance care planning at diagnosis and at each health and social care review”* (p. 14). Advanced decisions to refuse treatment can be made by individuals who are aged 18 or above with capacity. Advance decisions are recognised in the Mental Capacity Act [12] and should be written and witnessed, and must clearly stipulate the individual’s wishes regarding treatment in specific circumstances. Advance decisions to refuse treatment are legally binding, although individuals with capacity can change their mind and revoke the advanced decision [15]. Additionally, a Health and Welfare Lasting Power of Attorney enables individuals to nominate one or more people as “attorneys” who have the power to make decisions on their behalf should they be unable to do so [16].

It is also possible for medical professionals to withdraw treatment, for example discontinuing life support (ventilation) or removing feeding tubes, if this treatment is judged not to be in an individual’s best interests [17]. The withdrawal of treatment may also be referred to as “passive euthanasia” [18], which is lawful in the UK.

Debate regarding euthanasia is emotive and may involve strongly held, polarised opinions and be further confused by myriad terminology being utilised to define euthanasia [19]. Euthanasia can be defined as deliberately terminating life with the aim of alleviating suffering (this can be voluntary or involuntary) and assisted suicide can be defined as when an individual aids another person to take their own life (the individual must know the intention is to end life) [20]. At the time of writing, physician-assisted dying (euthanasia and physician assisted suicide) was illegal in the UK [20,21]

In England and Wales, the 1961 Suicide Act abrogated suicide and attempted suicide from being criminal acts; however, it remains a crime if an individual:

*“Does an act capable of encouraging or assisting the suicide or attempted suicide of another person, and the act was intended to encourage or assist suicide or an attempt at suicide”.*

[22]

In the UK, the debate regarding assisted dying continues and, in 2021, Baroness Meacher tabled a Private Member’s Bill in the House of Lords entitled “A Bill to enable adults who are terminally ill to be provided at their request with specified assistance to end their own life; and for connected purposes” [23]. Baroness Meacher states in the bill:

*“It would give terminally ill, mentally competent people over the age of 18 the right to choose the manner and timing of their death. To be eligible for an assisted death, two independent doctors would have to confirm that the person requesting assistance had a life expectancy of no more than six months.”*

[23].

At the time of writing, the bill was progressing through the UK Parliamentary process.

Baroness Meacher’s bill is only applicable to England and Wales. However, Liam McArthur (Member of the Scottish Parliament) successfully petitioned in 2022 to introduce the “Proposed Assisted Dying for Terminally Ill Adults (Scotland) Bill” [24].

An alternative position is presented by faith groups and other organisations, such as Care not Killing (2023) [25], which oppose changes to the law and promote palliative care. Opponents of euthanasia and assisted dying, as well as citing religious or moral grounds, highlight concerns around the abuse of legislation, individuals being coerced or pressured into ending their lives, and the stigmatisation of people with disabilities [25].

Recognising individuals and/or their families may request assistance or advice, healthcare professional bodies have issued guidance regarding assisted dying, for example the Royal College of Nursing (2016) [26], the General Medical Council (GMC) (2015) [27], and the British Medical Association (BMA) (2019) [28]. Several organisations have also recently moved to a neutral stance on assisted dying; The Royal College of Surgeons England in 2023 [29], the BMA in 2021 [30], and The Royal College of Physicians in 2020 [31].

Although professionals have been consulted regarding euthanasia and assisted dying, public opinion has been less canvassed [19]. A survey conducted by Pentaris and Jacobs (2020) [19] sought the public opinion regarding views about the legalisation of assisted dying and assisted suicide in cases of painful terminal illness and painful incurable, but not terminal, illness. The researchers note, overall, in the case of painful terminal illness, the majority of respondents were in favour of assisted dying and assisted suicide being legalised. However, there were very clear differences between ethnic groups, with respondents from minority ethnic backgrounds (Black/African/Caribbean, Asian, Other, Mixed/multiple ethnicity, Gypsy/traveller) being less in favour than white respondents. Furthermore, individuals with no religion were more likely to be in favour than respondents with a faith. In the case of non-terminal illness, the majority of respondents were not in favour, although ethnic and religious differences remained. While the survey was small (n = 297), the conclusions propose the public should be consulted by policy makers.

There are also examples of recent opinion polls, for example a YouGov poll in 2021 found 73% of respondents were in favour of assisted dying for terminally ill individuals who had capacity when approved by two doctors [32]. A poll of the British public by My Death My Decision, with the National Centre for Social Research (2019) [33], explored medically assisted dying for people with Alzheimer’s Disease, who still had capacity, with 77% of respondents stating this was always or sometimes acceptable.

However, the public opinion polls/surveys highlighted above related to individuals with “mental capacity;” as dementia progresses and cognitive impairment worsens, individuals lose capacity and are not deemed capable to make decisions. A survey of attitudes towards assisted dying and the withholding of life-sustaining treatment for PWD [34] found, in cases of severe dementia, those polled were generally in favour of being allowed to die (72.9%) of euthanasia by family or friend (50.9%) and physician-assisted suicide (59.5%). Moreover, there were smaller but still-significant percentages of respondents who were in favour of the above options in cases of mild and moderate dementia. Similar to Pentaris and Jacobs (2020) [19], differences emerged between ethnic groups, with white respondents being more in favour than respondents from minority ethnic backgrounds. Additionally, the respondents were more likely to agree to assisted dying and the withholding of life-sustaining treatment for themselves than for a partner.

While opinion polls are useful in identifying a general view, they are problematic in so far as polls only provide a superficial snapshot in time. This is particularly evident when gauging the opinion on nuanced issues, for example, Government policy during the COVID19 pandemic; “*Polling data only offer one simple perspective and do not illustrate the ambivalence many people feel around lockdown policies*” ([35], p. 1). Thus, for a complicated, emotive, and divisive issue such as euthanasia, polls have limited utility.

Whereas, a review of the literature exploring attitudes towards euthanasia for PWD reported, in general, PWD and carers were supportive of assisted dying in early dementia more than in advanced dementia. However, the public were more supportive of assisted dying, including in cases of advanced dementia, especially if the PWD had an advanced decision. Healthcare professionals were generally less accepting of assisted dying, although more were in favour if a PWD had made an advanced decision [36].

Given the ongoing debate in the UK regarding proposed legislative reforms to the law on assisted dying and a lack of information regarding public opinion, about both assisted dying and euthanasia, we set out to capture views by focusing one section of a Mass Observation Project Directive on dementia and euthanasia for PWD. The questions relating to euthanasia were part of a larger set of questions relating to dementia.

## 2. Method

The Mass Observation Project captures data from a panel of 500 members of the British public via a set of written questions (known as Directives) three times per year [37]. Respondents, known as “Observers”, respond to the questions in writing with no limit on the length of response and no obligation to respond to all or any questions. All Observers remain anonymous; therefore, responses are often frank and reflect highly personal perspectives, yielding a wealth of data. As a research team, we have found utilisation of Mass Observation Directives to be an efficient and effective approach to obtaining large amounts of data from representatives of the British public (see [38] for more information) and so commissioned a Directive which explored public perceptions of dementia and included the following questions regarding end of life:

- If you had a diagnosis of severe dementia, how would you wish to be cared for at the end of your life?
- What are your views about Euthanasia and dementia?

The Directive did not provide a definition of euthanasia. This paper reports responses to the second question.

### 2.1. Sample and Characteristics

A total of 143 Observers responded to the directive, and of these, 107 addressed the questions relating to euthanasia. Not all Observers provided demographic data, information for those who did is reported below.

#### 2.1.1. Gender

A total of 69.9% identified as female and 30.1% identified as male.

#### 2.1.2. Age

Mean age females = 60.7 years with an age range from 22 to 89. Mean age males = 57.6 with an age range from 34 to 89.

#### 2.1.3. Marital Status

A total of 58.1% married or in a civil partnership, 17.2% single, 11.8% widowed, 4.3% divorced, and 8.6% cohabiting.

Data analysis was an iterative process involving the research team, commencing with a high-level analysis, which led to conference presentations. Questions posed by and feedback from attendees resulted in further reflection on the data and prompted ongoing analysis. The final in-depth analysis was primarily conducted by two members of the research team who utilised the NVivo software package [39] to analyse data thematically, following the approach developed by Braun and Clarke 2022 [40]. The substantive codes and themes were discussed with and reviewed by other members of the research team to promote rigour. The aim was to develop themes which meaningfully encapsulated the data. Sections of data were coded (labelled) before integrating the codes into themes. Analysis revealed the following key themes arising from the data:

- Capacity;
- Legislation;
- Agency and Personal Philosophies;
- Disquietude;
- Incumbrance.

Representative quotations from Observers’ responses are provided for each theme. Each Observer has been assigned an alpha numeric pseudonym, e.g., O1, O2. Observers’ gender and age are provided where available. We begin with capacity as this emerged as a key concern and is pertinent to current debate related to legislative policy and procedure.

## 3. Findings

### 3.1. Capacity

Capacity was a fundamental issue which emerged for Observers who held a range of attitudes, values, and beliefs related to euthanasia. Crucially, there was evidence capacity and the ability to make informed decisions impacted Observers’ opinions to euthanasia. Indeed, there was evidence for some Observers their stance towards euthanasia was conditional on an individual having capacity:

*“I have no problems with euthanasia in principle—I think people should have control over their bodies and lives (providing sufficient safeguards are in place to stop abuse). However when people cannot make reasoned decisions themselves I do think that this should not be an option”.*

O1, Male 39.

*“I’m not sure where I stand on euthanasia in general… I’m fully aware dementia can be a very distressing and debilitating condition but I’m not sure it qualifies for euthanasia, especially as the person concerned could have little cognitive input to the decision.”*

O2, Female, 39.

The following Observer reflects how the issue of capacity impacts an individual’s autonomy and agency, highlighting the ethical dilemma faced regarding euthanasia for PWD:

*“By its very nature, dementia would make it difficult for anyone to make an informed and rational decision to end their life so this is a very murky area ethically!”*

O3, Female 64.

Although, during the early stages of dementia, individuals have the capacity to make decisions, for some Observers, the fact PWD eventually lose capacity precludes euthanasia in cases of dementia. Nevertheless, as indicated by the following quote, the issue of capacity is complicated and fluid, particularly during early stages of dementia; therefore, deciding at what point to act may be problematic:

*“But being demented means I couldn’t choose. I would uphold anyone’s right to end their own life whenever they wished, but with a mental condition, judging when the time is right is tricky.”*

O5, Female, 67.

Timing emerged as a key concern should euthanasia/assisted dying become available for PWD. The dilemma of balancing making a decision to hasten death too early against the possible loss of capacity and personal agency was recognised as a key variable affecting decision making. The following Observer was strongly in favour of euthanasia/assisted dying but faced the reality they may have to end their life earlier than may be strictly necessary:

*“I don’t believe in any magical gods, my life belongs to me and it’s up to me when I end it…I’ll have to make sure I do it while I’m still relatively healthy which seems a bit of a shame.”*

O6, Female, 38.

Capacity was a key factor in Observers’ accounts and influenced their attitude and beliefs about euthanasia. It was evident, for some Observers, the inability to make informed decisions prevented euthanasia from being an option for PWD. Consequently, the timing of decisions and when to act emerged as an ethical dilemma; the following quote encapsulates this:

*“If I had significant warning about encroaching severe dementia and therefore capacity to act I believe I would be interested in considering euthanasia were it available in the UK although it would be difficult to know at what point to act.”*

O7, Female, 44.

### 3.2. Legislation

As highlighted earlier, while there is ongoing debate regarding assisted suicide and euthanasia, both were unlawful at the time of the Directive and the time of writing. Observers indicated an awareness of the legal issues and attempts to change the law:

*“I’m sure it’s not legal in this country to make a decision to end someone else’s life”*

O3, Female, 63.

Some Observers also declared membership of organisations which supported and lobbied for legal changes:

*“I firmly support euthanasia and belong to Dignity in Dying: it is what I wish for myself if I was either demented or in great pain. I keenly support Lord Falconer’s bill and of course hope it will become law in time for me to take advantage of it”.*

O8, Female, 87.

There was also recognition the issue of changing UK law to permit euthanasia provoked polarised debate with strongly held views on both sides. The following Observer succinctly encapsulated the challenges to introducing euthanasia, as well as sharing doubt a resolution would be reached in the near future:

*“Euthanasia is a horrible word but there is no point in shying away from it. There are huge problems to be overcome if it should ever be introduced in this country—public opinion, religion, the medical profession, the ‘slippery slope’ and ‘life is sacred’ arguments, etc. However, I feel that it will have to be faced one day, although probably not in my lifetime.”*

O9, Male, 78.

Indeed, the following Observer expressed frustration with Parliament for a lack of action regarding euthanasia, while acknowledging perhaps no UK Government would be willing to make it lawful:

*“I believe that the government and those who can make changes to the right to die, shy away from the subject. Nobody seems to want to take responsibility for such a massive decision about giving people the right to choose how they die where possible. But somebody has to, too many people are carrying on with their lives in pain, in deep sorrow or frustration or anger, who desperately want to die but who are being kept alive because that’s what doctors see as their duty of care, to keep people alive.”*

O10, Female, 64.

A solution regarding the political impasse was suggested by the following Observer:

*“So many people feel this way (in favour of euthanasia) and yet we are denied our wish. Why?? Surely with the correct procedures in place it could be managed? How about a referendum on it, especially for the elderly??”*

O11, Female, 65.

However, Observers highlighted a major barrier to legalising euthanasia is concern about misuse and individual motivations, which the following Observer uses as an explanation of Government caution regarding changes to legislation:

*“…but it would be great if euthanasia was legal here. I guess the powers to be do not trust the public to be honest about the reasons for exiting.”*

O12, Male, 67.

The possibility of the abuse and/or misuse of euthanasia was a frequent issue in Observers’ responses and a commonly expressed opinion was, should euthanasia be introduced, it would need to be strictly controlled and regulated. Concern was expressed regarding malign motivations for encouraging or pressuring an individual to agree to end their life. Observers wrote about a number of such concerns, including coercion:

*“I also think there should be strong safeguards to protect the patient from exploitation and to determine that the decision is wholly their own and not forced upon them.”*

O13, Female, 25.

Financial gain for individuals who stood to inherit on someone’s death was raised:

*“On the other hand, if euthanasia became legal then there is obviously huge scope for wrongdoing, bumping people off for their inheritance etc. So I am confused as to the best way to go on that.”*

O14, Female, 72.

Concerns regarding money were not limited to individual gain. The quote below highlights uneasiness financial considerations for the state, such as the cost or other consequences of individuals requiring extensive levels of health and social care, may result in euthanasia:

*“It would worry me if it got to the stage in society when people with dementia were “bumped off” because they were a drain on resources.”*

O15, Female, 71.

The question of who should decide on ending an individual’s life and when was also raised in relation to medical professionals:

*“I don’t think doctors should be given the right to euthanase people. It is playing God and for some it will end very badly; they will feel their loved one has been “bumped off” much too early, when there was still life in them. I can totally imagine that at the top of the news bulletin. Because people think carers are bad with the abuses some of them carry out, letting people be euthanised would make it so much worse”.*

O16, Female, 29.

There was evidence Observers were aware of the legislative issues relating to euthanasia and they acknowledged challenges faced by legislators. Indeed, a number expressed concerns regarding how euthanasia would be regulated if it were to become legal in the UK, in particular how misuse and abuse of the law could be prevented.

### 3.3. Agency and Personal Philosophies

Nevertheless, irrespective of the law, there was evidence some Observers had a strong personal philosophy regarding euthanasia. When asked for opinions on euthanasia for PWD, a minority of Observers expressed clear views without any justification for their statement:

*“And I do believe in euthanasia. And that’s all I want to say.”*

O17, Female, 34.

*“I’m agin (against) it.”*

O18, Male, 85.

While the Observers quoted above simply stated a view, the majority expanded their responses and offered explanations for their support or opposition to euthanasia with “principles” emerging as a key factor. One approach was the proposal individuals’ agency should be respected and this extends to autonomy regarding their death, and thus choice and the option of euthanasia should be available:

*“My views on Euthanasia are that we old people should be able to end our lives when we are ready. If we have lived a rich, full life with our families and have fulfilled our time on earth why not?”*

O11, Female, 85.

*“I think everyone deserves the right to determine the fate of their own lives.”*

O13, Female, 25.

However, some Observers denied agency in relation to their death, expressing a faith-based belief their lives belonged to a deity who would decide when their lives would end:

*“I have a strong Christian faith, and that influences my views on euthanasia. I believe that the time for me to die is decided by God and is not my decision to make.”*

O19, Female, 50.

Having a philosophically based belief regarding death was also evident for individuals with secular principles. The following Observer indicated strong beliefs regarding euthanasia/assisted dying:

*“I am a firm believer in assisted dying. I believe in the Humanist principle that dying is part of living and is therefore something we would all wish to do well and with dignity and pride. If we need assistance during this period then we should be able to receive it. And if this means that we want help to die we should be able to receive it.”*

O20, male, 41.

Although the following Observers would not choose euthanasia for themselves, there was an acknowledgement it might be something others may wish to explore and a recognition of individual choice, reinforcing the importance of personal agency:

*“I can never condone euthanasia for myself although it would be impudent of me to judge what other people accept.”*

O21, female, 79.

*“I really wouldn’t want to go down that route. I respect the right of those who do choose that way, and it is a brave decision for all involved.”*

O22, male, 48.

Personal philosophy and associated “principles” may result in strong views regarding euthanasia for PWD, and euthanasia per se. Indeed, this was evidenced above. However immutable principles, whether pro- or anti-euthanasia, may detract from reasonable debate, resulting in inertia and the political impasse regarding euthanasia discussed in the theme “Legislation” above.

### 3.4. Disquietude

The dementia prognosis of cognitive deterioration and what this entails in terms of dignity, personhood, and agency triggered a desire from a number of Observers to be able to end their lives should they develop dementia, although this was mainly associated with the severity of the disease and its impact on individuals:

*“I have talked about getting dementia and I honestly do not want to live if I have severe dementia. I would be quite happy to be euthanised if I no longer knew people or where I was etc, and I had no quality of life mentally”*

O23, female, 57.

The concept of personhood and whether this is linked to cognition, especially memory, was reiterated by the following Observer who, again, indicated being able to remember one’s life history and know and interact with others was a crucial aspect of life:

*“If I was no longer myself and have no memories of my family or my own life I do not see the point of keeping me alive if I was unable to breathe or function for myself.”*

O24, male, 49.

Evidence of dementia provoking fear also emerged from Observers’ narratives. The following Observers wrote about what receiving a diagnosis of dementia meant to them and how they believed they would react:

*“If I had a diagnosis of severe dementia I would like to be able to take a pill and die. I have no wish to live with dementia it must be a terrifying experience to not know where or who you are”.*

O25, female, 76.

Other Observers expressed consternation regarding how dementia might affect them as the disease progressed:

*“For me losing my mind or memory is my worst nightmare. I wouldn’t want to get to the stage where I can’t recognise my husband and son. I also wouldn’t want them to see me in that way either…With regards to euthanasia I don’t get why this isn’t made legal in the UK”.*

O37, female, 50.

Concern regarding needing comprehensive assistance from others and the possible indignity of requiring personal care was another factor which prompted support for euthanasia:

*“To have to be fed, washed and supervised at the toilet is the most unimaginably dreadful scenario for me. If I thought that I could end my life before I reached that stage I would be greatly reassured.”*

O35, male, 75.

A range of concerns about dementia were expressed by Observers which encompassed all stages of the disease, from diagnosis to end of life. Some Observers articulated a sense of dread regarding dementia, indicating they would prefer death rather than the loss of personhood and dignity which they might experience as the dementia worsened. The issue of needing care and being reliant on others also emerged from Observers’ accounts and is explored below.

### 3.5. Incumbrance

The notion of becoming, or the potential to become, an incumbrance on family or carers was a concern. Observers recognised euthanasia as an option to mitigate a perceived impact of dementia on a range of other people. The following quotes express a general concern about incumbering other individuals or the state:

*“I wouldn’t want to be a burden on anyone, so I would choose to die earlier of something else.”*

O28, female,46.

*“And just as I am a firm believer and supporter of the hospice movement, so I always believe strongly that if we are faced with terminal illness, being a burden on loved ones or the state, or if on our own, left for our care to the kindness of strangers, we should have the right to say how and when we end our lives.”*

O10, female,64.

Moreover, other Observers had more specific concerns, for example writing about wider family and friends:

*“I would not want to be a burden to family or friends and as such I believe in euthanasia. There is no cure and once advanced past a certain stage, no quality of life. This is not living and most people I know feel the same”*

O29, female, 50;

Their children:

*“My husband makes his view known to everyone. “If my wife dies, I want to go with her. No way to do I want to burden my children”. He is emphatic about it!!”*

O11, female, 85;

Or their spouse:

*“I think both of us would hate to be a burden on the other, and as such would seriously contemplate assisted dying. However, I think in practice the remaining healthy partner might feel uneasy about this, and as such the dementia sufferer would probably end up in a care home.”*

O30, male, 49.

Some Observers indicated their concern about incumbrance emanated from personal experience of illness:

*“I try not to be a burden… and often do too much, then suffer with exhaustion.”*

O31;

And witnessing the stress and strain faced by carers:

*“I feel quite strongly that if I became affected in this way I would like my end to be with euthanasia, I would not like my family to have the burden of caring for me, because I have seen what this situation can do to families.”*

O32, female, 74.

There was also recognition incumbrance was not restricted to emotional pressure but could also impact one financially:

*“I am very clear that if I had a cast iron diagnosis of severe dementia I would be quite happy to end my life early. I would not wish to be a burden to my family either emotionally or financially and I would try to make sure that my life was ended in as tidy a way as possible.”*

O33, male, 41.

Observers frequently expressed concerns regarding incumbering others, especially close family and friends. Observers who commented about burden were mainly concerned with not burdening others and related this to choosing euthanasia. Nevertheless, being a burden was not always perceived as being a sufficient reason to end one’s life:

*“I think until I lost capacity I would want to remain alive- when I become scared, or am unable to have a good quality of life I would like the option of euthanasia but on MY terms not because I’m a burden to someone.”*

O34.

## 4. Discussion

This paper contributes to the wider conversation taking place in the UK regarding euthanasia and, given the lacuna in the data relating to this for PWD, offers valuable insight from members of the British public. Before going on to discuss the findings, it is important to note we did not provide Observers with a definition of euthanasia. This facilitated Observers interpreting euthanasia within their own frame of reference. Commonalities in their accounts emerged and these resonated with both the ongoing discussion in the UK about euthanasia and previous research. Moreover, the Observers did not ask for any clarification of the term “euthanasia” and there was evidence they were aware this was illegal in the UK, with some indicating they were aware of attempts to change legislation.

Whilst accounts were provided from Observers who were pro, against, and unsure about euthanasia for PWD; one issue was common across the three categories, i.e., “capacity”. As found in other research (inter alia [36]), capacity was a key conundrum and some Observers proposed, because of cognitive decline and the concomitant loss of capacity, euthanasia is not appropriate for PWD. Conversely, others indicated the loss of capacity and severe cognitive impairment was a reason for their pro-euthanasia stance. It must be acknowledged the issue of capacity impacting the ability to make informed decisions does present a challenge regarding euthanasia. Indeed, the Assisted Dying Bill currently progressing through the UK Parliament [23] requires individuals making a declaration they wish assisted dying to be:

- “mentally competent” when making a declaration, and be;
- “reasonably expected to die within six months”.

Unlike ACPs, declarations under the bill are legally binding; therefore, an individual can make their wishes known whilst they have capacity to do so. However, given the life expectancy for PWD is difficult to gauge, for example individuals diagnosed with Alzheimer’s disease may live between 8 and 10 years after their diagnosis [41], it is problematic for doctors to confirm an individual’s life expectancy. Furthermore, an individual may lose capacity well in advance of their expected time of death and thus be unable to make a declaration under the bill. It can be argued the timescales within the bill remove the personal agency of individuals with dementia.

However, euthanasia where individuals lack capacity has been addressed in the Netherlands, where a test case before the Supreme Court ruled “A physician may carry out a written request beforehand for euthanasia in people with advanced dementia” [42]. Similarly, in Canada, Medical Assistance in Dying is permitted where individuals who previously made a written arrangement to waive final consent have lost capacity, unless they clearly demonstrate refusal or resistance [3]. It is conceivable, therefore, should Baroness Meacher’s bill become law in the UK, it may evolve to consider the issue of capacity and euthanasia for people with advanced dementia. Nonetheless, as it stands, the UK bill appears to have limited utility in cases of dementia. Some Observers expressed strong opinions regarding euthanasia both for PWD and euthanasia per se; responses also indicated Observers had considered the legal and moral implications of making euthanasia lawful in the UK. Observers are members of the general British public; therefore, this suggests a wider conversation for which specifically canvassed public opinion would be welcomed. However, it must be noted Observers are mainly from white, middle-class backgrounds and previous research suggests differences in views about euthanasia between individuals of different ethnic backgrounds. Individuals from minority ethnic backgrounds were generally less in favour of euthanasia, as were individuals with a faith [9,34]. Any consultation of the public must therefore ensure views are obtained from as wide a sample of the population as possible and the views of individuals from diverse ethnic and faith backgrounds are captured. In May 2023, the UK Government launched an inquiry into assisted dying/assisted suicide. However, although 317 written responses were received from within and without the UK, it appears circa two thirds of responses originated from politicians, academics, medical and legal professionals, and a range of organisations such as charities and religious institutions [43]. Moreover, as the individuals did not provide any demographic information, it is not possible to comment on the diversity of the sample. Consequently, we hypothesise the data captured were not representative of individual lay members of the public.

The misuse of euthanasia and individuals being coerced to end their lives emerged as a key issue for Observers who held a range of views about euthanasia. Many Observers proposed robust safeguards must be included in legislation regarding euthanasia. The absence of robust legislative safeguards has the potential to leave individuals exposed to undue influence, coercion, or abuse. Indeed, Wand et al. (2019) [44] highlight, when developing and enacting assisted dying legislation, the “recognition and mitigation of risks of such abuse” (p. 79) is critical. The Assisted Dying Bill [23] makes a clear provision regarding coercion and duress, with two doctors having to certify an individual freely made a declaration regarding ending their life. Additionally, the doctors are required to discuss alternatives such as palliative care with the patient. Although it should be recognised, it may be difficult to be certain an individual has not been subject to coercion and is acting freely. Observers frequently expressed a desire not to be a “burden” on others, i.e., partners, family, and friends. However, given the crisis in social care in the UK, appropriate care provision may not be available or individuals may not have financial resources to fund such care. Moreover, individuals with sufficient funding may be concerned about the impact on the inheritance for their families. Consequently, care for individuals may fall to partners or family, contributing to the feeling of being an incumbrance, which may influence decisions regarding euthanasia.

## 5. Conclusions

The Observers’ accounts reflected a range of views about euthanasia and dementia, which were thoughtful, at times emotive, and well expressed. It was evident, therefore, many Observers had considered this issue previously. Pentaris and Jacobs (2020) [19] suggested policy makers should consult the British public regarding legislation in this area, and the findings of our study support this proposal.

## 6. Limitations

Whilst the Mass Observation Project is undoubtably a useful approach to obtain the views of members of the British public, it does have limitations. There is a lack of heterogeneity in the sample, with the majority of Observers being white, middle aged or older, and female. Consequently, there is a lack of data from individuals with minority ethnic backgrounds, which is particularly problematic for this paper given the evidence of ethnicity related differences in attitudes to euthanasia highlighted above. However, given the homogeneity in the sample, a range of views were expressed.

Observers are free to write as much or as little as they wish, often resulting in rich data; however, it is not possible to return to Observers for clarification of their response or for further information, which is possible when capturing qualitative data via interviews. It is essential, therefore, to ensure Directive questions are clear and unambiguous. Should we commission a further Directive regarding euthanasia and dementia, we would refer to the most recent bill and specifically explore views regarding assisted suicide.

## Data Availability

Data is available from the Mass Observation Project Archive.
